# Social observation modulates the influence of socioeconomic status on pro-environmental behavior: an event-related potential study

**DOI:** 10.3389/fnins.2024.1428659

**Published:** 2024-08-09

**Authors:** Bowei Zhong, Nana Niu, Jin Li, Yun Wu, Wei Fan

**Affiliations:** ^1^CAS Key Laboratory of Behavioral Science, Institute of Psychology, Chinese Academy of Sciences, Beijing, China; ^2^Department of Psychology, University of Chinese Academy of Sciences, Beijing, China; ^3^Department of Psychology, Hunan Normal University, Changsha, China; ^4^Cognition and Human Behavior Key Laboratory of Hunan Province, Changsha, China; ^5^Institute of Interdisciplinary Studies, Hunan Normal University, Changsha, China

**Keywords:** SES, pro-environmental behavior, social observation, event-related potentials, N2, N400, P3

## Abstract

Understanding the psychological antecedents of socioeconomic status (SES) on pro-environmental behavior is crucial for effectively encouraging individuals from different socioeconomic backgrounds to address environmental issues. Previous research has separately examined the influence of SES and social observation on pro-environmental behavior. However, little is known about whether social observation moderates the influence of SES on pro-environmental behavior, and the underlying neurophysiological mechanisms remain uncharacterized. Using event-related potential (ERPs), we adopted the green purchase paradigm and manipulated subjective SES, to examine whether the influence of SES on pro-environmental behavior is moderated by social observation. The behavioral results revealed that individuals of high SES tended to purchase more eco-friendly products under the observable condition than those in the non-observable condition. The ERP results revealed that participants with high SES exhibited more negative N2 and N400 amplitude during environmental decisions in the non-observable condition than in the observable condition, indicating that high SES individuals experience less cognitive conflict during environmental decisions, which may reflect the attenuated cost–benefit trade-off due to reputational incentives in the presence of observers. Additionally, individuals with high SES exhibited greater reputational motivation when observed, as indicated by larger P3 amplitude. However, these differences were not observed among individuals with low SES. These findings suggest that SES is associated with distinct psychological and behavioral differences in pro-environmental behavior, moderated by social observation, evident across both the early and later stages of environmental decisions in the brain.

## 1 Introduction

Human activities, including water, energy use, and climate change, are driving the sixth mass extinction, resulting in a high proportion of biodiversity loss and the extinction of different species (Giannetti et al., 2023; Nangia et al., 2023). To effectively mitigate environmental degradation, there is an increasing emphasis on individuals from diverse socioeconomic backgrounds engaging in pro-environmental behavior (Chen et al., 2023; Niu et al., 2023). Previous studies have explored the influence of SES on pro-environmental behavior. For example, social scientists and economists suggest that engaging in green consumption serves as a signal of belonging to the higher SES (Kennedy and Givens, 2019), indicating that the higher SES individuals are associated with more pro-environmental intentions (Grandin et al., 2022; Niu et al., 2023; Sun et al., 2023). However, some research suggests that individuals in the higher SES do not always engage in pro-environmental behavior (O'Connor et al., 2002; Marquart-Pyatt, 2012; Lo, 2016; Blankenberg and Alhusen, 2019). Given the divergent research outcomes mentioned above, how SES influences pro-environmental behavior remains unclear, and the neurocognitive mechanisms underlying pro-environmental behavior remain unexplored.

Importantly, previous functional magnetic resonance imaging (fMRI) studies have consistently shown that prosocial behavior (e.g., pro-environmental behavior) involves activation in three main sets of brain regions: (i) empathy-related regions like the anterior insula (AI) and temporoparietal junction (TPJ), indicating empathic concern associated with prosocial behavior; (ii) reward-related regions such as ventromedial prefrontal cortex (vmPFC), and ventral striatum (*VS*), suggesting that prosocial behavior is driven by reward; (iii) cognitive control-related regions including the dorsolateral prefrontal cortex (dlPFC) and dorsal anterior cingulate cortex (dACC), indicating that individuals inhibit selfish motives (Hu et al., 2017; Cui et al., 2022; Davis et al., 2023; Li et al., 2023b). Considering that pro-environmental behavior as a specific applied example of prosocial behavior, aligning with long-term environmental goals, may share similarities with other forms of prosocial behaviors (e.g., helping behavior) (Barclay and Barker, 2020; Farrelly and Bhogal, 2021; Li et al., 2023b), it is worth considering whether prosocial behavior motivation may be specific, applicable to a particular type of behavior (e.g., altruistic behavior), or domain-general, indicating that the same motivation can apply to pro-environmental behavior. Notably, the event-related potentials (ERPs) technique has provided important information regarding the temporal properties of decision processing, which can elucidate the temporal dynamics of individual motivation processing in pro-environmental behavior. However, little attention has been paid to the neurocognitive mechanisms underlying pro-environmental behavior, particularly the temporal dynamics of neural activity in the brain.

Numerous studies have shown that event-related potentials (ERPs) offer a high temporal resolution (Carlson et al., 2016; Zubair et al., 2020; Li et al., 2023b), which can be used to implicitly measure the brain’s dynamic neural response during decision processing (Li et al., 2023a). Existing studies involving prosocial decision (e.g., environmental decision) mainly focus on the dynamic neural responses of N2, N400 and P3 components (Li et al., 2020b, 2022, 2023b; Jing et al., 2022). The early component of decision processing is N2, a negative wave peaking between 200 and 350 ms after stimulus onset, which reflects attention resource allocation and cognitive control (Folstein and Van Petten, 2008; Gui et al., 2015; Li et al., 2022, 2023a). For example, recent researchers used a lottery-choosing task to investigate whether individuals exhibit differential prosocial behavior in the presence of others due to reputational concern. These results showed that when deciding whether to help acquaintances or strangers, individuals weigh the costs and benefits and allocate more cognitive resources to resolve conflict when there are no observers, thus eliciting more negative N2 amplitude (Zhan et al., 2020; Li et al., 2022, 2023a). The N400 is identified as the late negative component that typically emerges around 400 ms following stimulus presentation, particularly in the frontal and central regions of the scalp, which reflects cognitive and emotional conflicts during environmental decisions (Jing et al., 2022). For example, inducing empathy with nature elicits a smaller N400 amplitude during green purchasing decisions than the control condition, indicating that individuals experience less cognitive dissonance (Jing et al., 2022). In addition, the P3, typically peaking around 300 ms after stimulus onset in the central and parietal regions (San Martin, 2012), is associated with attentional resource allocation and cognitive control processing (Hu and Mai, 2021; Li et al., 2023b), and also reflects prosocial motivation (Carlson et al., 2016; Li et al., 2020a,b, 2022). Previous research regarding social decision processing has indicated that the results of P3 is not always consistent. For example, several researchers have found that larger P3 reflects greater attention resource allocation and cognitive control, suggesting that individuals invest more cognitive resources in cost–benefit calculations during decision-making (Li et al., 2018, 2023b; Hu and Mai, 2021). However, some studies indicate that P3 is associated with motivational level and affective evaluation (Carlson et al., 2016; Li et al., 2020a,b, 2022). When making charitable donations to highly empathetic organizations (Carlson et al., 2016; Hu and Mai, 2021), as well as when making prosocial decisions to benefit friends (Li et al., 2020b, 2022), larger P3 was elicited, suggesting the prosocial motivations. In addition, previous studies have shown that a larger LPP positively correlates with higher individual scores on altruistic personality traits (Zhan et al., 2020).

Notably, individuals from different socioeconomic backgrounds exhibit variations in social rewards, especially when making decisions in the presence of others, which may further influence the trade-off between self-interest and potential benefits. Pro-environmental behavior, a specific prosocial behavior, can also increase when reputational concern is made salient, such as being observed by others (Jung et al., 2018; Sangwan et al., 2024). Individuals are more inclined to donate money when they are observed by others, activating the ventral striatum (i.e., a part of the brain’s reward system) associated with social reward processing, similar to the monetary reward processing (Izuma et al., 2010; Bhanji and Delgado, 2014; Qu et al., 2019). Considering the cost-reward models of prosocial behavior, which propose that individuals calculate the costs and rewards of their actions to maximize benefits and minimize costs (Hu et al., 2017; Qu et al., 2019), it prompted us the following question: do individuals from different SES differ in their cost–benefit considerations? Costly signaling theory suggests that pro-environmental behavior in the public context signals one’s willingness and ability to afford substantial expenses, which is linked with higher social status (Griskevicius et al., 2010; Jung et al., 2018; Barclay and Barker, 2020; Farrelly and Bhogal, 2021). Therefore, the affluent may be more inclined to contribute to social welfare in the public context, whereas individuals from low SES may prioritize avoiding personal costs over seeking social approval (Kraus and Callaghan, 2016; Barclay and Benard, 2020). Most importantly, studies have found that social observation moderates the influence of SES on prosocial behavior, suggesting that individuals of high SES engage in more prosocial behaviors in the public context due to reputational concern and impression management (Kraus and Callaghan, 2016). However, few studies have explored the SES differences in pro-environmental behavior under reputational incentives. Based on the aforementioned findings, the current study aimed to further clarify two questions. Specifically, our first aim was to investigate whether individuals with high SES are motivated by reputational concern in the presence of others (Weininger and Lareau, 2009; Kraus and Callaghan, 2016; Kawamura and Kusumi, 2018; Roberts et al., 2021), thereby exhibiting more pro-environmental behavior. Second, in terms of methodology, it’s crucial to recognize the potential inconsistencies of SES on pro-environmental behavior, which may be attributed to the measurement method of self-report through both questionnaires and behavioral experiments, such as social desirability effect (Laidley, 2011; Meyer, 2015; Lange and Dewitte, 2019; Grandin et al., 2022; Niu et al., 2023). Therefore, the second aim was to implicitly examine whether social observation moderates the effect of SES on pro-environmental behavior and the underlying neural mechanism.

To address the above two questions, we conducted an ERP study to investigate the influence of SES and social observation on pro-environmental behavior at the electrophysiological level. Based on the above evidence, we hypothesized that social observation can moderate the influence of SES on pro-environmental behavior (Kraus and Callaghan, 2016; Kawamura and Kusumi, 2018), as reflected by the N2, N400 and P3 components. Especially, pro-environmental behavior refers to actions taken by individuals that benefit the environment, requiring attention resources allocated to weigh personal costs and environmental benefits (Lange et al., 2018; Barclay and Barker, 2020; Li et al., 2021, 2023b; Zhong et al., 2022; Niu et al., 2023), which can be motivated by the presence of others, especially for high SES individuals who are highly sensitive to reputational incentives and impression management (Griskevicius et al., 2010; Izuma et al., 2010; Kerekes, 2010; Kraus and Callaghan, 2016; Kawamura and Kusumi, 2018; Barclay and Barker, 2020; Farrelly and Bhogal, 2021; Yamawaki et al., 2023). Therefore, individuals of high SES, when in the presence of others, can prioritize reputational incentives, attenuating the cost–benefit weighing to gain a good reputation (Kraus and Callaghan, 2016), leading them to engage in cost–benefit calculations and elicit more negative N2 and N400 amplitudes only under non-observable conditions. In addition, individuals of high SES, when observed by others, may disguise their selfish interests for longer-term benefits (Zhan et al., 2022), reflected in the later stage of P3. Considering the lack of consensus in studies on decision processing in P3, we did not have a concrete prediction here. However, individuals of low SES, due to having fewer resources, may invest more cognitive resources in elaborated processing during environmental decisions, making them less likely to engage in pro-environmental behavior, which may reflect the P3 component.

## 2 Methods

### 2.1 Participants and experimental design

A prior power analysis for a 2 (SES: low SES vs. high SES) × 2 (social observation: observable vs. non-observable condition) mixed design was used to determine the sample size, using G*Power 3.1.9 (*F* tests, analysis of variance [ANOVA]: repeated-measures, within-between interaction, power = 0.90; effect size *f* = 0.25; α = 0.05) (Faul et al., 2007; Farrelly and Bhogal, 2021; Cui et al., 2022; Li et al., 2024). The results indicated that a minimum of 46 participants were required to achieve 90% statistical power. To ensure the reliability of results, 59 participants (20.17 ± 1.98 years) were recruited from Hunan Normal University, and were randomly assigned to either the high SES or low SES condition (both conditions of participants showed no difference in their preference for eco-friendly products and other potential confounding factors, see Supplementary material). Among them, 30 participants were randomly assigned to the high SES priming condition (15 females), while 29 participants were randomly assigned to the low SES priming condition (17 females). All participants were physically healthy, had no history of mental illness, were right-handed, and had a normal or corrected-to-normal vision. Before the experiment began, participants were informed of their rights according to the Declaration of Helsinki and provided written informed consent. This study received ethical approval from the Ethics Committee of Hunan Normal University (Ethics approval number: 2023330).

### 2.2 Experimental materials and procedure

#### 2.2.1 SES manipulation

**Figure 1:**
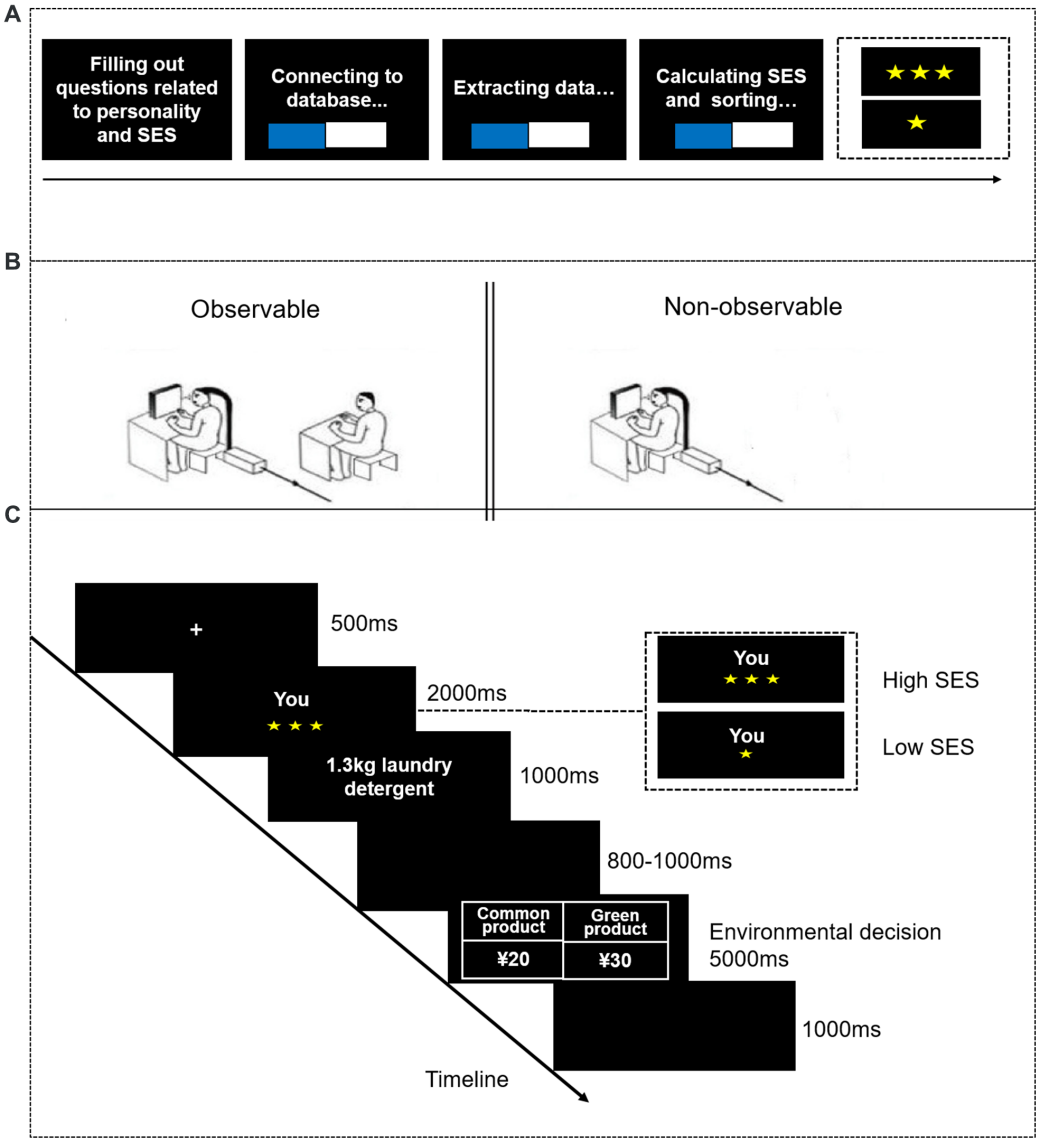
**(A)** The flowchart illustrates the socioeconomic status (SES) priming. **(B)** Schematic representation of the social observation manipulation (Li et al., 2022, 2023b). **(C)** The flowchart of a single trial depicts the environmental task. Initially, a fixation was presented for 500 ms, followed by the display of the participant’s relative position for 2,000 ms, which emphasized their hierarchy in either the high (i.e., three stars) or low SES (i.e., one star) position. Subsequently, the product type was presented for 1,000 ms, followed by a random blank screen (800–1,000 ms). Afterward, the decision interface was presented (5,000 ms). Participants were instructed to make decisions regarding common products (by pressing the “F” key) or eco-friendly products (i.e., green products) products (by pressing the “J” key).

Previous literature has found that brief priming of subjective SES can elicit behavioral patterns similar to those exhibited by individuals with long-term exposure to a specific SES (Kraus et al., 2013; Guo et al., 2015), making it feasible to manipulate subjective SES to explore its effect on pro-environmental behavior. To ensure the priming effect, our study adopted the feedback method to manipulate subjective SES (Greitemeyer and Sagioglou, 2016, 2018). Specifically, before the task commenced, participants were required to complete questions about their own and their parents’ SES and personality. They were informed that upon submission, the procedure would automatically connect to a database, developed by the professional database company we collaborated with, containing information on SES and the personality of university students nationwide. Subsequently, the procedure would automatically match and extract groups of university students nationwide whose personalities were similar to the participants. Then, the procedure would compute the SES values for each matched student. Finally, the participant would receive feedback regarding their SES level (see Figure 1A).

In addition, after the experiment, participants were asked to subjectively rate their subjective SES using the MacArthur Scale (1 = lowest hierarchy, 10 = highest hierarchy) in comparison to university students nationwide with similar characteristics (Adler et al., 2000). A higher score indicated a higher subjective SES.

#### 2.2.2 Social observation manipulation

The social observation manipulation was adapted from Li et al.’s study (2023), utilizing the presence of an observer. In the observable condition, a stranger (same-gender observer aged 24, whom the participants reported they had never seen before) sat discreetly behind the participant, positioned 50 cm to the right of the participant (see Figure 1B). Subsequently, the participants were informed that the observer, who was familiarizing themselves with the upcoming experimental task, would diligently record their environmental decisions. These decisions would then be communicated to a same-gender unfamiliar individual, who would later collaborate with the participants to complete a cooperative game (public goods game, PGG) after the experiment. Moreover, the participants were informed that the more cooperative both partners were, the more the reward they would receive, creating a reciprocal reputational concern (Barclay and Barker, 2020; Li et al., 2022, 2023b; Zhong et al., 2022). In the non-observable condition, the participants were informed that the observer was on their way and would arrive shortly (i.e., the sequence from non-observable condition to observation condition) or that the observer needed to engage in other unrelated tasks and would no longer continue observing (i.e., the sequence from observation condition to non-observable condition) (Izuma et al., 2010; Zhong et al., 2022), and were instructed to complete the task individually.

Additionally, to assess the effectiveness of the social observation manipulation, participants were asked to answer two questions: “When the observer was present, to what extent did you believe that the observer was diligently recording your decisions?” (1 = not at all, 9 = extremely believable) and “When the observer was not present, did you believe that your decisions were completely confidential?” (1 = not at all, 9 = completely confidential).

#### 2.2.3 Environmental task

The environmental task involved a total of 8 products, including a multifunctional keychain, 150-sheet 3-ply tissues, a 96-page A5 notebook, a portable phone stand, a transparent glass cup, 200 mL shampoo, a collapsible umbrella, and 1.3 kg laundry detergent. Specifically, each type of product was paired with both common and environmentally friendly alternatives. The prices of these 8 common products were set based on market surveys at 3.14 RMB, 5.15 RMB, 8 RMB, 10 RMB, 12 RMB, 15.12 yuan, 17.1 RMB, and 20 RMB, respectively. Taking into account the premium pricing of eco-friendly products, the prices were set at 25, 50, 75, 100, 125, 150, and 175% higher than those of common products (Zhong et al., 2022; Li et al., 2023a; Niu et al., 2023).

Before the experiment, participants were informed about the reward for participating: a basic participation fee (¥ 20) and an additional participant fee (¥ 55). To ensure participants’ commitment in each trial, they were informed that, after the experiment, one trial would be randomly selected to calculate the final additional participant fee, which was determined as the additional participant fee of 55 minus the product expenditure (the price of the corresponding product purchased in one randomly selected trial). Therefore, the total participation fee equaled the basic fee plus the final additional participant fee (additional participant fee—product expenditure) plus the corresponding product.

Upon commencement of the experiment, the participants sat comfortably in an electromagnetic shielding room approximately 75 cm from a desktop computer monitor (23.8-in LED screen; refresh rate: 60 Hz; resolution: 1,920 × 1,080 pixels). Stimulus presentation and data acquisition were performed using E-prime 2.0 (Psychological Software Tools, Inc., Pittsburgh, PA). As illustrated in Figure 1C, in each trial, the participants were first presented with a blank screen for 500 ms, followed by a display indicating their SES through the number of stars (i.e., three stars indicating high SES, one star indicating low SES) for 2,000 ms. Next, the product type, such as “200 mL shampoo,” was shown for 1,000 ms. After a blank screen (800–1,000 ms), the prices of both common and eco-friendly products were displayed for 5,000 ms, during which participants were instructed to make environmental decisions by pressing keys (“F” for common products, and “J” for eco-friendly products). Participants took 6 trials to familiarize themselves with the formal experimental. The formal experiment consisted of two blocks, one for the observable condition and another for the non-observable condition, with counterbalancing between participants. Each block comprised 56 trials, with 8 products each having 7 prices.

In conclusion, the participants were initially randomly assigned to either the high SES or low SES priming group. Subsequently, they completed the environmental task under the observable or non-observable conditions. Finally, manipulation checks were conducted for both SES and social observation.

#### 2.2.4 EEG recording and analysis

Continuous electroencephalograph (EEG) signals were recorded using 64 Ag/AgCl scalp sites embedded in an elastic cap according to the international 10–20 EEG/ERP System (ANT Neuro, Enschede, Netherlands). The online reference electrode was CPz. Additionally, the electrooculogram (EOG) was captured from four electrodes positioned laterally to each eye and above and below the right eye. Electrode impedances were consistently maintained below 5 kΩ at every recording site. The EEG signals were band-pass filtered between 0.05 and 100 Hz, and sampled at a rate of 500 Hz per channel. After completing continuous EEG recording, offline data processing was conducted to remove artifacts thoroughly. The data were re-referenced to the average of the left and right mastoid electrodes, and were band-pass filtered between 0.1 and 30 Hz. Independent component analysis (ICA) was employed to remove eye blinks and motion artifacts (Delorme and Makeig, 2004). Artifacts exceeding ±80 μV were excluded from averaging (Zhan et al., 2020; Yan et al., 2023; Zhong et al., 2023). Table 1 displays the remaining artifact-free trials used for the ERP analysis after preprocessing, in which were no significant differences observed across experimental conditions (*Fs* < 0.5, and all *Ps* > 0.05). Epochs for analysis were set from 200 ms before to 800 ms after the onset of the environmental decision presentation (Carlson et al., 2016; Zhan et al., 2020; Xu et al., 2023).

**Table 1:** The trials under each condition (mean ± standard deviation).

	Observable condition	Non-observable condition
High SES	52.57 ± 4.52	52.77 ± 3.81
Low SES	53.14 ± 3.42	52.52 ± 3.16

Combined with previous ERP findings and visual observation of topographical maps of the entire brain (Carlson et al., 2016; Zhan et al., 2020; Li et al., 2023b; Jing et al., 2022), the mean amplitudes of the N2 (290–390 ms) and N400 (450–550 ms) were analyzed for 6 electrode sites (F3, Fz, F4, FC3, FCz, FC4) in the frontocentral region respectively, and the mean amplitude of P3 (350–450 ms) was analyzed for 6 electrode sites (CP3, CPz, CP4, P3, Pz, P4) in the central-parietal regions. In conclusion the ERP data were analyzed as the mean amplitudes for the chosen electrode sites in each condition. In addition, behavioral and ERP data were subjected to a repeated-measures analysis of variance (ANOVA) of 2 (social observation: observable, non-observable condition) × 2 (SES: high SES, low SES), and the analysis was conducted using SPSS 23.0 (IBM Corp., Armonk, NY, United States). The Greenhouse–Geisser method was utilized to correct the *p*-values for main and interaction effects due to violations of the sphericity assumption, while Bonferroni corrections were applied for multiple comparisons at *p* < 0.05. The effect size of the ANOVA was reported as partial eta-squared ( $\eta_{p}^{2}$ ). The effect size thresholds for $\eta_{p}^{2}$ are 0.01 for small, 0.06 for medium, and 0.14 for large. For Cohen’s d, the thresholds are 0.2 for small, 0.5 for medium, and 0.8 for large (Cohen, 1988; Zhang et al., 2023).

## 3 Results

### 3.1 Manipulation checks

#### 3.1.1 Subjective SES manipulation check

An independent samples *t*-test was conducted on participants’ self-rated subjective SES scores. The results revealed significant differences in subjective SES, *t*(57) = 13.47, *p* < 0.001, Cohen’s *d* = 3.52. Participants in the high SES priming condition (mean ± standard deviation: 7.30 ± 1.56) rated themselves significantly higher hierarchy than those in the low SES priming condition (2.48 ± 1.15), indicating the effectiveness of the SES manipulation.

#### 3.1.2 Social observation manipulation check

In the social observation condition, a one-sample *t*-test was conducted, revealing that participants believed the observer was recording their decisions during observation (6.46 ± 1.95), which significantly differed from the median value of 5, *t*(58) = 5.74, *p* < 0.001. In the non-observable condition, the result revealed that participants perceived their decisions to be more confidential in the non-observable condition (6.54 ± 1.95) than the median value of 5, *t*(58) = 6.07, *p* < 0.001. These findings suggest that the manipulation of social observation was successful.

### 3.2 Behavior results

#### 3.2.1 Pro-environmental behavior

A repeated measures analysis of variance (ANOVA) revealed a significant main effect of SES, *F*(1, 57) = 31.62, *p* < 0.001, $\eta_{p}^{2}$ = 0.36 (Table 2). Participants in the high SES condition (0.47 ± 0.17) chose significantly higher proportions of eco-friendly products than those in the low SES condition (0.22 ± 0.17). Additionally, there was a significant main effect of social observation, *F*(1, 57) = 7.94, *p* = 0.007, $\eta_{p}^{2}$ = 0.12, which indicated that participants in the observable condition (0.36 ± 0.18) chose more eco-friendly products compared to the non-observable condition (0.33 ± 0.17).

**Figure 2:**
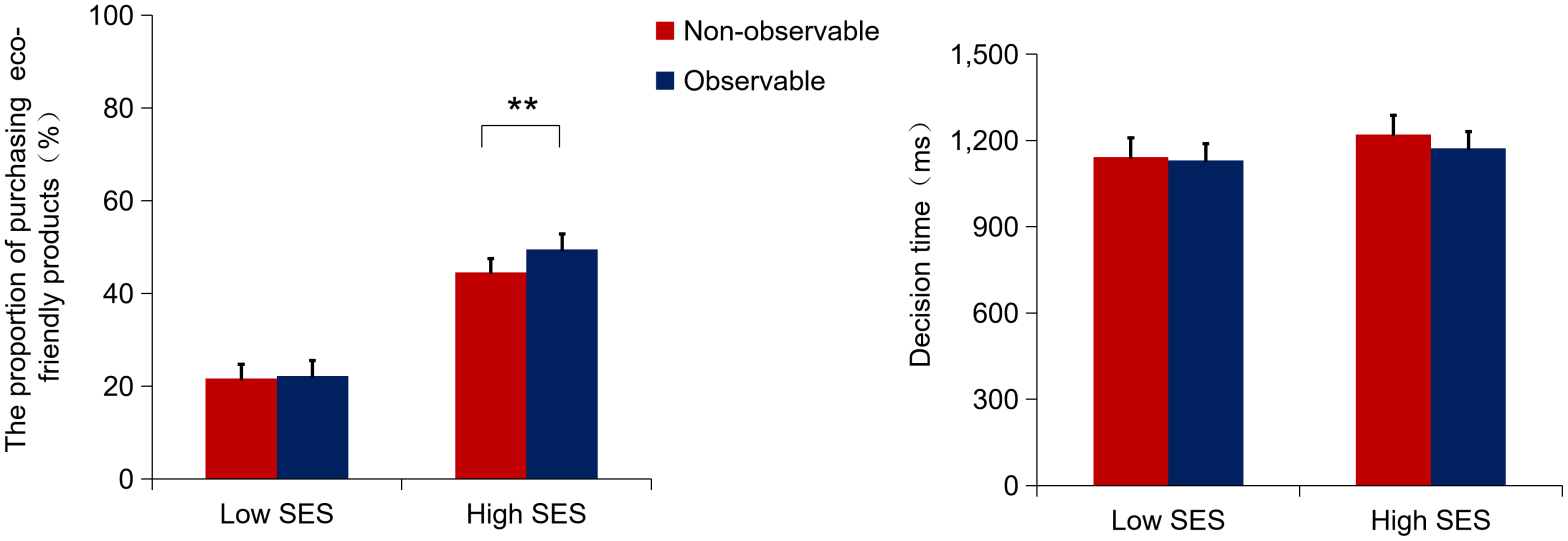
The proportion of purchasing eco-friendly products under observable and non-observable conditions across SES. **p < 0.01, SES, socioeconomic status. Error bars indicate the standard error of the means (SE).

Furthermore, there was a significant interaction between SES and social observation, *F*(1, 57) = 5.23, *p* = 0.026, $\eta_{p}^{2}$ = 0.08. Simple effects analysis revealed that in the high SES condition, the proportion of eco-friendly products was significantly higher in the observable condition (0.50 ± 0.21) than in the non-observable condition (0.45 ± 0.19), *F*(1, 29) = 9.85, *p* = 0.004, $\eta_{p}^{2}$ = 0.25. However, in the low SES condition, there was no significant difference in the proportion of eco-friendly products between the observable condition (0.22 ± 0.15) and the non-observable condition (0.22 ± 0.14), *F*(1, 28) = 0.22, *p* = 0.65 (see Figure 2). The other analysis including price was detailed in the Supplementary material, which found that the interaction effect between SES and social observation persists reliably after including price in the analysis.

**Table 2:** Summary of the behavioral and ERP results with corresponding *F* values (*p* values).

	The proportion of eco-friendly products	Decision times	N2	P3
SES	**31.62 (<0.001)**	0.55 (0.463)	0.16 (0.695)	**6.45 (0.014)**
Social observation	**7.94 (0.007)**	0.38 (0.379)	1.50 (0.226)	1.90 (0.174)
SES × social observation	**5.23 (0.026)**	0.30 (0.59)	**8.31 (0.006)**	**6.91 (0.011)**

Significant effects are marked in bold. SES, socioeconomic status.

#### 3.2.2 Decision times

The main effect as well as the interaction effect were insignificant.

### 3.3 ERP results

#### 3.3.1 N2 (290–390 ms)

**Figure 3:**
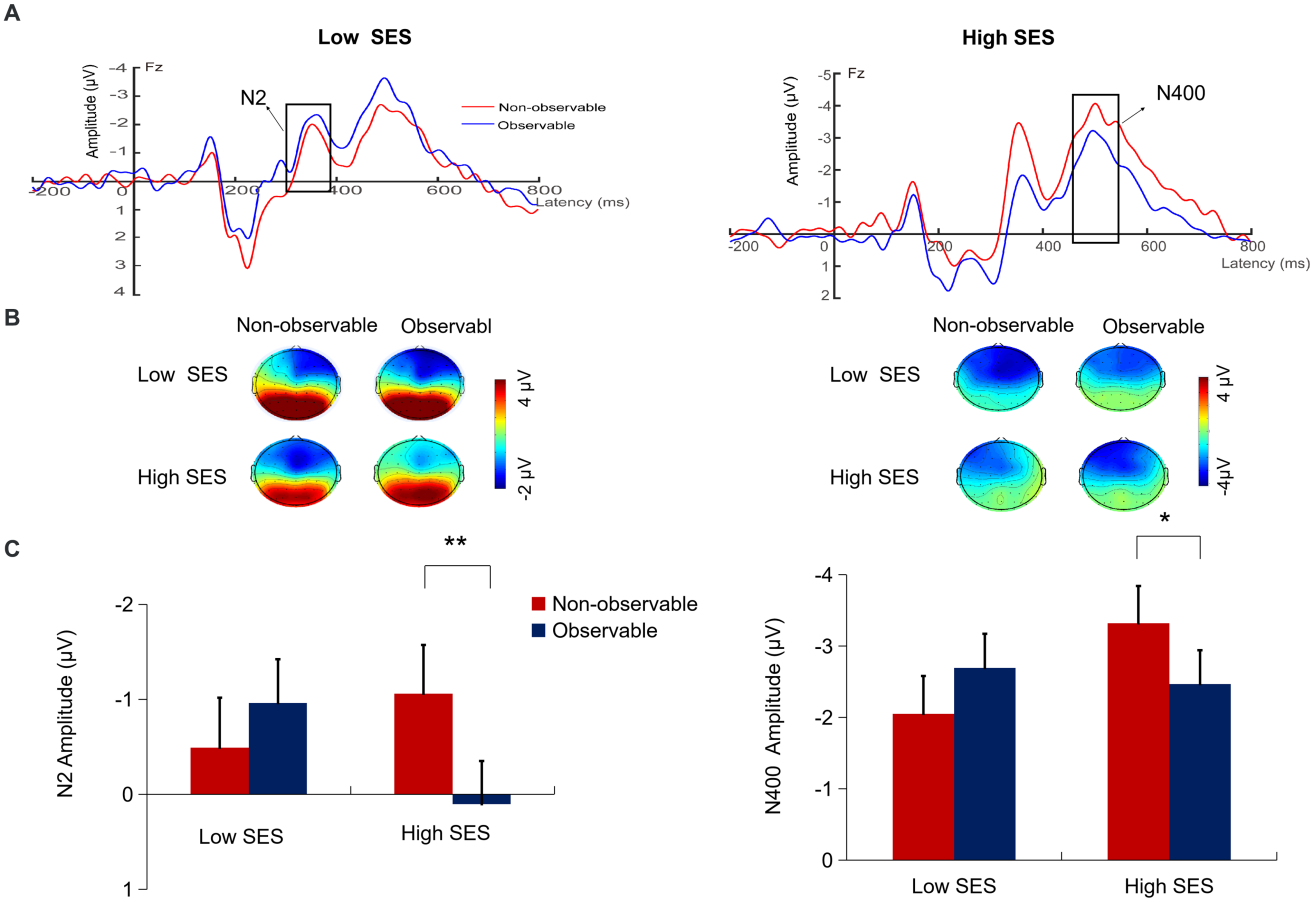
**(A)** Grand-average ERPs waveforms at the Fz. **(B)** Topographic map of N2 (290–390 ms) and N400 (450–550 ms) for each condition. **(C)** The mean amplitudes of N2 and N400 for each condition. *p < 0.05, **p < 0.01. Error bars indicate the standard error of the means (SE).

A repeated measures ANOVA revealed that the interaction between SES and social observation was significant, *F*(1, 57) = 8.31, *p* = 0.006, $\eta_{p}^{2}$ = 0.13. Simple effects analysis revealed that in the high SES condition, the N2 amplitude under observable condition (0.10 ± 2.53 μV) was more positive than that in the non-observable condition (−1.06 ± 2.98 μV), *F*(1, 29) = 12.12, *p* = 0.002, $\eta_{p}^{2}$ = 0.30. However, in the low SES condition, there was no significant difference in the N2 amplitude under observable condition (−0.96 ± 2.43 μV) compared to the non-observable condition (−0.49 ± 2.64 μV), *F*(1, 28) = 1.04, *p* = 0.317. No other effects were significant (*p*s > 0.05) (see Figure 3).

#### 3.3.2 P3 (350–450 ms)

A repeated measures ANOVA showed a significant main effect of SES, *F*(1, 57) = 6.45, *p* = 0.014, $\eta_{p}^{2}$ = 0.10. Participants in the low SES condition (3.43 ± 1.99 μV) exhibited larger P3 amplitudes during environmental decisions compared to those in the high SES condition (2.13 ± 1.94 μV).

**Figure 4:**
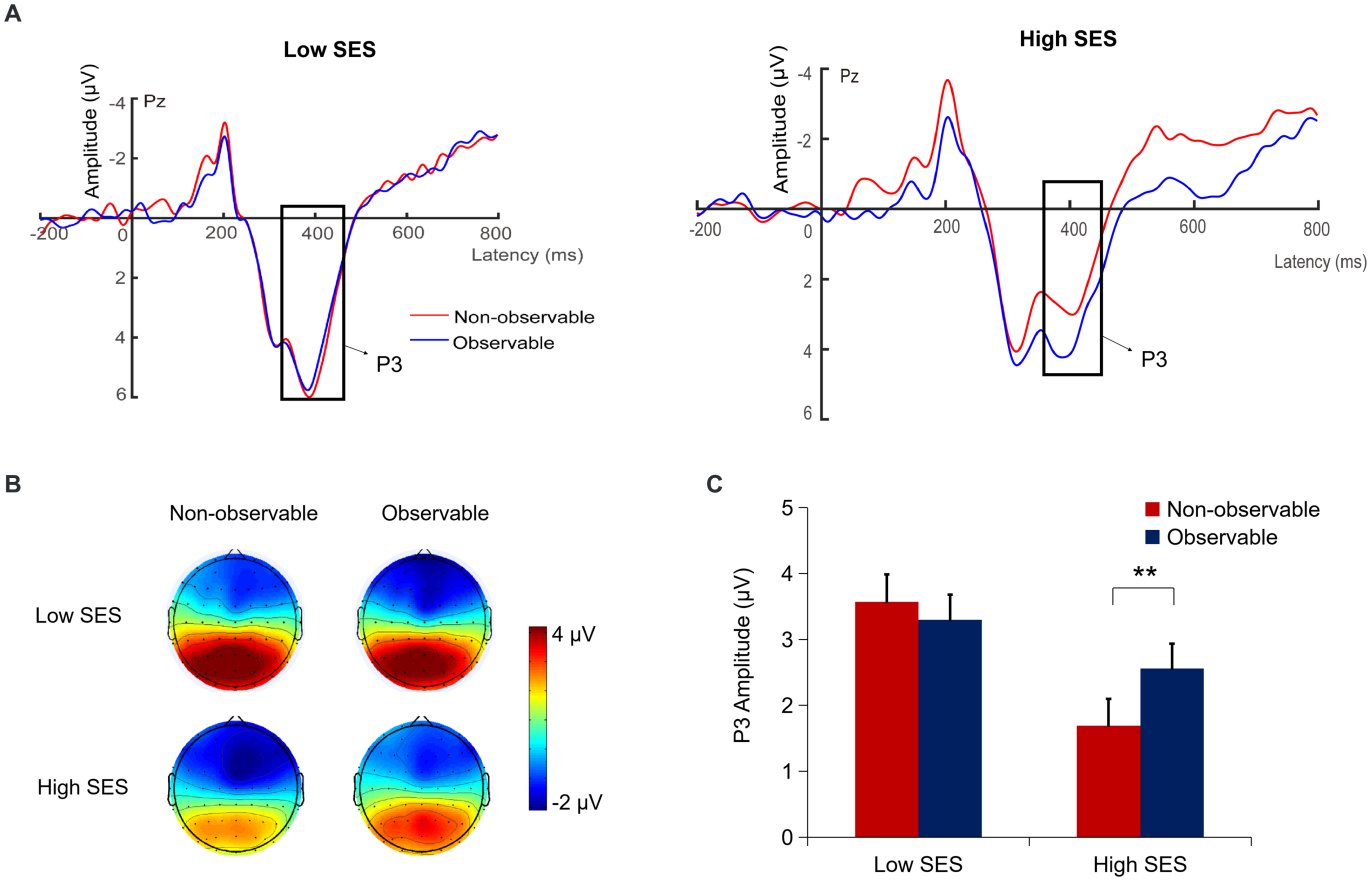
**(A)** Grand-average ERPs waveforms at the Pz. **(B)** Topographic map of P3 (350–450 ms) for each condition. **(C)** The mean amplitudes of P3 for each condition. **p < 0.01. SES, socioeconomic status. Error bars indicate the standard error of the means (SE).

Importantly, there was a significant interaction between SES and social observation, *F*(1, 57) = 6.91, *p* = 0.011, $\eta_{p}^{2}$ = 0.11. Simple effects analysis revealed that in the high SES condition, the P3 amplitude under the observable condition during environmental decisions (2.56 ± 2.03 μV) was significantly larger than that in the non-observable condition (1.69 ± 2.00 μV), *F*(1, 29) = 12.32, *p* = 0.001, $\eta_{p}^{2}$ = 0.30. However, in the low SES condition, there was no significant difference in the P3 amplitude under the observable condition during environmental decisions (3.30 ± 2.08) compared to the non-observable condition (3.57 ± 2.46), *F*(1, 28) = 0.57, *p* = 0.456. No other effects were significant (*p*s > 0.05) (see Figure 4).

#### 3.3.3 N400 (450–550 ms)

A repeated measures ANOVA revealed that the interaction between SES and social observation was significant, *F*(1, 57) = 6.03, *p* = 0.017, $\eta_{p}^{2}$ = 0.10. Simple effects analysis revealed that in the high SES condition, the N400 amplitude under observable condition (−2.47 ± 2.84 μV) was more positive than that in the non-observable condition (−3.32 ± 2.74 μV), *F*(1, 29) =4.59, *p* = 0.041, $\eta_{p}^{2}$ = 0.14. However, in the low SES condition, there was no significant difference in the N400 amplitude under observable condition (−2.69 ± 2.29 μV) compared to the non-observable condition (−2.05 ± 2.98 μV), *F*(1, 28) = 1.93, *p* = 0.176. No other effects were significant (*ps* > 0.05) (see Figure 2).

## 4 Discussion

This study is the first attempt to explore the influence of SES and social observation on pro-environmental behavior using the neurophysiological approach. Consistent with our hypothesis, these results indicate that, when considering the personal costs and potential benefits of pro-environmental behavior, individuals from high SES are motivated by social reputation, engaging in more pro-environmental behavior, as evidenced by larger P3, smaller N2 and N400 in the presence of one observer. Conversely, individuals from low SES, constrained by limited resources, prioritize their self-interests over social reward, decreasing their tendency to engage in pro-environmental behavior regardless of whether they are being observed. These findings suggest that social observation moderates the influence of SES on pro-environmental behavior, as reflected in both early and later stages of neural response in the brain.

### 4.1 Social observation moderates the influence of SES on the proportion of eco-friendly products

Behavioral results showed that individuals with high SES purchased more eco-friendly products compared to those with low SES. Furthermore, we observed that in the presence of others, individuals with high SES purchased more eco-friendly products than the non-observable condition, which was not found in the low SES condition. These findings align with prior research, indicating that individuals with high SES demonstrate a greater inclination toward prosocial behavior to maintain a favorable reputation when others are present (Kraus and Callaghan, 2016). Pro-environmental behavior has social signaling value, displaying personal positive characteristics such as social status and trustworthiness (Barclay and Barker, 2020; Vesely et al., 2020; Farrelly and Bhogal, 2021; Zhong et al., 2022), which can enhance individuals’ reputation and status, making them more appealing social partners when observed by others (Kraus and Callaghan, 2016; Brick et al., 2017; Barclay and Barker, 2020; Vesely et al., 2020; Farrelly and Bhogal, 2021). Therefore, the presence of others promotes individuals to suppress selfishness and engage in pro-environmental behavior. Furthermore, reputation mechanisms vary among individuals from different SES (Kraus and Callaghan, 2016). Especially, individuals with high SES are more inclined to contribute to social welfare in the public context, exhibiting stronger prosocial motivations for reputational concern and impression management (Kraus and Callaghan, 2016; Vesely et al., 2020; Zhong et al., 2022). Whereas individuals from low SES lacking in resources may prioritize avoiding perceived costs over seeking social approval (Kraus and Callaghan, 2016; Barclay and Benard, 2020). Based on these theories and empirical research, individuals with high SES tend to engage in more pro-environmental behavior when observed by others, as it incurs personal costs.

### 4.2 Social observation moderates the neural responses of SES on pro-environmental behavior

In the ERP results, we observed the differences in neural response related to pro-environmental behavior between individuals from different SES in both the early and later stages under observable and non-observable conditions. Specifically, in the early N2 component, our study found that individuals with high SES elicited more negative N2 amplitude under the non-observable condition than the observable condition. A more negative N2 reflects individuals investing more attentional resources in weighing costs and benefits (Li et al., 2022, 2023a). In the late N400 component, consistent with the N2 results, our study found that high SES individuals, when observed by others compared to when not observed, exhibited a more positive N400 component, which indicate that individuals with high SES invested more attentional resources in environmental decisions under non-observable conditions. However, for individuals with low SES, there was no difference in their behavior regardless of whether others observed them. These results are consistent with previous research indicating that N2 is associated with cognitive resources allocated to cost–benefit calculation (Kraus and Callaghan, 2016; Kawamura and Kusumi, 2018; Li et al., 2022, 2023a) and N400 reflects the cognitive and emotional conflicts experienced by individuals during environmental decisions (Jing et al., 2022). Therefore, these findings suggest that at the early semi-automatic stage and later stage of environmental decisions, individuals of high SES, influenced by the presence of others, attenuate the deliberate calculation of self-interests and environmental benefits due to reputational incentives, thereby inducing less cognitive conflict and negative emotional conflict during environmental decisions.

More importantly, during the later stage of environmental decision processing, our study found that social observation modulates the influence of SES on pro-environmental behavior, as reflected in the P3 component. Individuals from low SES exhibited greater P3 amplitude when making environmental decisions. These results suggest that individuals with low SES emphasize the importance of weighing costs and benefits and investing more cognitive resources to resolve dilemmas. These findings are consistent with previous research showing that P3 is associated with resource allocation and cognitive control (Li et al., 2018, 2023b; Hu and Mai, 2021). As mentioned above, pro-environmental behavior as domain-specific prosocial behavior, aligning with long-term environmental goals, shares similarities with other forms of prosocial behaviors (e.g., altruistic behavior) (Barclay and Barker, 2020; Farrelly and Bhogal, 2021; Li et al., 2023b), which refers to actions taken by individuals that benefit the environment, requiring attention resource allocation to weigh personal costs and environmental benefits (Lange et al., 2018; Barclay and Barker, 2020; Li et al., 2021; Zhong et al., 2022; Li et al., 2023b; Niu et al., 2023). Therefore, considering the premium associated with eco-friendly products (Niu et al., 2023), individuals from low SES prioritize weighing costs and benefits, investing more attentional resources and cognitive effort in environmental decisions.

Conversely, our findings showed that individuals with high SES demonstrated larger P3 amplitude when observed by others compared to when not observed, which was not found among individuals with low SES. The larger P3 also reflects greater motivational significance associated with prosocial behavior when making decisions (Carlson et al., 2016; Li et al., 2020b, 2022). These results indicate that individuals with high SES are influenced by reputational incentives when observed, disguising their self-interest and exhibiting greater prosocial motivation. These findings extend previous behavioral research by showing that individuals with high SES engage in prosocial behavior driven by reputational management in the public context. Especially, individuals of high SES, when observed by others, strategically activate their social reputation motives and prompt them to adjust pro-environmental behavior to maintain a favorable personal image among others for future benefits as others tend to be more cooperative toward them (Barclay and Barker, 2020; Vesely et al., 2020; Koundouri et al., 2023), and thus choose selfish altruistic behavior for longer-term benefits (Zhan et al., 2022). These findings suggest that during the later stage of decision processing, individuals from high SES exhibit greater pro-environmental motivation in the presence of others to maintain a positive self-image. In contrast, individuals from low SES, constrained by limited resources, may have reduced capacity to resist short-term interests, leading to pro-environmental behavior that is not influenced by the presence of others.

In conclusion, pro-environmental behavior, as a domain-specific form of prosocial behavior, reflects individuals’ positive attributes and involves weighing the costs against the environmental benefits (Barclay and Barker, 2020; Li et al., 2023b). Participation in pro-environmental behavior may be motivated by the desire to uphold one’s reputation when under observation, potentially resulting in greater future rewards. Notably, it is also worth noting that motivational disparities in pro-environmental behavior (e.g., self-interest and reputational concerns) exist across different SES. Moving forward, techniques such as functional magnetic resonance imaging (fMRI) or functional near-infrared spectroscopy (fNIRS) can be employed to further elucidate activation patterns in the corresponding brain regions associated with different motives in information integration and value computation.

## 5 Implications and future directions

This study holds academic significance and practical implications. Through event-related potential (ERP) technology, we investigated the neural processing of pro-environmental behavior in individuals of different SES when observed by others, confirming distinct psychological and behavioral characteristics across SES at the electrophysiological level. These findings enhance our understanding of how SES influences pro-environmental behavior, providing valuable insights for promoting such behavior across different SES. Therefore, businesses should adequately identify the psychological needs of target consumer groups throughout the entire life cycle of green products. Specifically, for consumers with high SES, marketing strategies such as eco-labels should be utilized to highlight the altruistic attributes of pro-environmental behavior, as these individuals can be driven by reputational incentives and impression management. Additionally, for consumers from low SES, the premium associated with eco-friendly products should be taken into account. Efforts should be made to keep the prices of green products comparable to or even lower than those of common products.

However, our study also had some limitations that justify further investigation. On the one hand, it is essential to recognize that this study involves student samples, and should be caution exercised when generalizing the results to broader populations. On the other hand, this study built upon prior research and categorized SES into high and low hierarchies (Kraus et al., 2009; Eom et al., 2018; Sherman et al., 2022), unveiling SES disparities within the context of Chinese culture at the electrophysiological level. However, it is noteworthy that SES can also be further stratified into high, middle, and low hierarchies. Future research is warranted to investigate the difference in pro-environmental behavior among individuals from high, middle, and low SES, which can provide a comprehensive understanding of whether and how to promote pro-environmental behavior across different SES.

## 6 Conclusion

This study investigates the neural responses underlying different psychological and behavioral mechanisms of SES on pro-environmental behavior, moderated by social observation, across both early and later stages of environmental decisions. Individuals with high SES, when observed by others, demonstrate heightened pro-environmental motivations driven by reputational incentives. Conversely, individuals from low SES, facing resource constraints, may prioritize immediate personal interests, leading to less engagement in pro-environmental behavior regardless of the presence of others.

## Data availability statement

The data that support the findings of this study are available on request from the corresponding author.

## Ethics statement

The studies involving humans were approved by The Ethics Committee of Hunan Normal University (Ethics approval number: 2023330). The studies were conducted in accordance with the local legislation and institutional requirements. The participants provided their written informed consent to participate in this study.

## Author contributions

BZ: Conceptualization, Visualization, Writing – original draft, Writing – review & editing, Formal analysis, Investigation, Methodology. NN: Conceptualization, Methodology, Data curation, Visualization, Writing – original draft, Writing – review & editing, Investigation. JL: Conceptualization, Funding acquisition, Methodology, Supervision, Writing – review & editing. YW: Writing – review & editing. WF: Conceptualization, Formal analysis, Supervision, Writing – original draft, Writing – review & editing.
