# Supplementary material for: Social observation modulates the influence of socioeconomic status on pro-environmental behavior: an event-related potential study
Source: Front Neurosci. 2024 Aug 9;18:1428659. doi: 10.3389/fnins.2024.1428659 (PMC11341474; doi:10.3389/fnins.2024.1428659)
Supplement: Supplementary file 1 [file Data_Sheet_1.docx]

**Supplementary materials:**

Social observation modulates the influence of socioeconomic status on pro-environmental behavior: An event-related potential (ERP) study

A questionnaire was administered before the experiment to control for potential differences in objective socioeconomic status (SES) among individuals from different SES backgrounds. It also aimed to control for confounding factors between the two SES priming conditions that might influence pro-environmental behavior. The questionnaire included items measuring parents' educational level, preference for eco-friendly products, environmental concern, connection to nature, biospheric values, and perceived social norms.

Specifically, regarding objective SES, previous research has found that education, rather than income or employment status, exerts a stronger influence on pro-environmental behavior and is associated with increased awareness and interest in environmental issues (Blankenberg & Alhusen, 2019). In this study, the educational level of parents was assessed among a homogeneous sample of college students by providing participants with the following options to choose from (1) elementary school or below, (2) junior high school, (3) high school/secondary vocational school/technical school, (4) junior college/night school/radio and television university, (5) undergraduate, (6) master's degree or above. The highest level of education attained by both parents was then selected (Bai et al., 2021; Chen et al., 2022; Tan & Kraus, 2015).

What’s more, to evaluate participants' **preference toward eco-friendly products**, two items were utilized: 'Buying eco-friendly products is important to me' and 'I often purchase eco-friendly products.' Participants rated these statements using a 7-point scale, ranging from 1 for 'strongly disagree' to 7 for 'strongly agree'. Higher scores indicate a more favorable attitude toward purchasing eco-friendly products (α = 0.77) (Roux et al., 2015).

This study also employed the Chinese version of the New Ecological Paradigm Scale (NEP), revised by Wu et al. (2012), to measure participants' **environmental concerns** (Dunlap et al., 2000). The scale includes both the New Ecological Paradigm and the Dominant Social Paradigm, and participants rated their agreement using a 5-point Likert scale, ranging from 1 for 'strongly disagree' to 5 for 'strongly agree'. Items related to the Dominant Social Paradigm were reverse-scored. The average of all item scores was calculated, with higher scores indicating greater environmental concern (α = 0.78).

**The Inclusion of Nature in the Self** (INS) scale was developed by Schultz to measure participants' connection to nature. This scale consists of a single item depicting 7 pairs of circles, with one representing the self and the other representing nature. The degree of overlap between the two circles ranges from 1 for 'no overlap at all' to 7 for 'nearly complete overlap'. Participants were asked to choose the pair that best represents their relationship with nature. A greater overlap between the two circles indicates a stronger connection between individuals and nature (Schultz, 2002).

In assessing **biospheric values**, four items were utilized to measure participants' attitudes toward environmental protection and pollution prevention (Liu & Wu, 2013). Participants rated these items on a 5-point Likert scale, ranging from 1 for 'not important at all' to 5 for 'very important'. The average score of these four items was calculated to represent biospheric values, with higher scores indicating stronger biospheric values (α = 0.96).

In assessing **perceived social norms**, a single item was employed to measure participants' perceptions of the proportion of their society who engage in pro-environmental behavior (i.e., perceived descriptive social norms). Participants were asked: "What percentage of people do you think engage in environmentally friendly behaviors in your society, such as carpooling or saving energy? Please fill in the percentage from 1 to 100 (Eom et al., 2016).

In conclusion, a *t*-test was conducted to explore whether there were differences in various dimensions between participants exposed to different SES priming conditions.

*1.1 Results for different scales*

The main effects of SES were insignificant for all measured variables: education level, *t*(57) = 1.24, *p* = 0.221; preference for eco-friendly products, *t*(57) = 0.58, *p* = 0.564; environmental concern, *t*(57) = 1.12, *p* = 0.270; connection to nature, *t*(57) = 0.24, *p* = 0.811; biospheric values, *t*(57) = 0.17, *p* = 0.870; and perceived social norms, *t*(57) = 0.70, *p* = 0.485.

Table 1 Means and standard deviations of different SES individuals on each scale

|  | High SES | Low SES |
| --- | --- | --- |
| Education level | 2.97 ± 1.25 | 2.62 ± 0.86 |
| The preference for eco-friendly products | 2.82 ± 0.80 | 2.98 ± 1.34 |
| Environmental concern | 3.88 ± 0.39 | 4.01 ± 0.47 |
| Connection to nature | 4.73 ± 1.46 | 4.83 ± 1.56 |
| Biospheric values | 4.48 ± 0.54 | 4.46 ± 0.68 |
| Perceived social norms | 54.23 ± 24.21(*%*) | 49.69 ± 25.47(*%*) |

*1.2 Results of costs on the proportions of eco-friendly products*

We further conducted a 2 (SES: low SES vs. high SES) × 2 (social observation: observable vs. non-observable condition) ×7 (costs: 25%, 50%, 75%, 100%, 125%, 150%, 175%) repeated measures analysis of variance (ANOVA) on the proportions of eco-friendly products. The main effect of costs was significant, *F*(6, 52) = 146.43, *p* < 0.001, η= 0.72; multiple comparisons showed that individuals were less inclined to purchase eco-friendly products as the personal cost increased(See Table 1 for detailed results). The main effect of SES was significant, *F*(1, 57) =31.49, *p* < 0.001, η= 0.36. Participants in the high SES condition (0.47 ± 0.17) showed significantly higher proportions of eco-friendly products than those in the low SES condition (0.22 ± 0.17). Additionally, there was a significant main effect of social observation, *F*(1, 57) = 7.67, *p* = 0.008,$\text{η}_{p}^{2}$=0.12, which indicated that participants in the observable condition (0.36 ± 0.18) chose more eco-friendly products compared to the non-observable condition (0.33 ± 0.17).

Table 1. The proportion of green products purchased at different personal costs

| The price of eco-friendly products  higher than common products | *M* | *SD* |
| --- | --- | --- |
| 25% | 0.74 | 0.28 |
| 50% | 0.54 | 0.32 |
| 75% | 0.38 | 0.29 |
| 100% | 0.27 | 0.26 |
| 125% | 0.20 | 0.23 |
| 150% | 0.17 | 0.18 |
| 175% | 0.14 | 0.16 |

Importantly, the interaction between SES and costs was significant, *F*(6, 52) = 4.37, *p* < 0.05, η= 0.07. Subsequently, simple effect analysis was carried out and the main effect of SES was significant at seven different personal costs in the present study (at 25% price level: *F*(1, 57) = 15.87, *p* < 0.001, η= 0.22; at 50% price level: *F*(1, 57) =23.42, *p* < 0.001, η= 0.29; at 75% price level: *F*(1, 57) = 32.13, *p* < 0.001, η= 0.36; at 100% price level: *F*(1, 57) = 20.99, *p* < 0.001, η= 0.27; at 125% price level: *F*(1,57) = 18.69, *p* < 0.001, η= 0.25; at 150% price level: *F*(1, 57) = 19.70, *p* < 0.001, η= 0.26; at 175% price level: *F*(1, 57) = 13.53, *p* = 0.001, η= 0.19 (see Fig.1).

Fig.1. SES differences in the proportion of eco-friendly products across different price levels. Error bars indicate the standard error of the means (SE).

In addition, there was a significant interaction between SES and social observation, *F*(1,57) = 5.85, p =0.019, η= 0.09. The other main and interaction effects were insignificant, with *p*s > 0.05.

**References**

Bai, J., Yang, S. L., Xu, B.X., Guo, Y.Y. (2021). How can successful people share their goodness with the world: The psychological mechanism underlying the upper social classes’ redistributive preferences and the role of humility. *Acta Psychologica Sinica, 53*(10), 1161-1172.

Blankenberg, A.-K., & Alhusen, H. (2019). On the determinants of pro-environmental behavior: A literature review and guide for the empirical economist. *Center for European, Governance, and Economic Development Research (CEGE)*(350).

Chen, S.J., Yang, S.S., Wang, H., & Wan, F.H. (2022). Subjective social class positively predicts altruistic punishment. *Acta Psychologica Sinica, 54*(12), 1548-1561.

Dunlap, R. E., Van Liere, K. D., Mertig, A. G., & Jones, R. E. (2000). New trends in measuring environmental attitudes: measuring endorsement of the new ecological paradigm: a revised NEP scale. *Journal of Social Issues, 56*(3), 425-442.

Eom, K., Kim, H. S., Sherman, D. K., & Ishii, K. (2016). Cultural Variability in the Link Between Environmental Concern and Support for Environmental Action. *Psychol Sci, 27*(10), 1331-1339.

Roux, C., Goldsmith, K., & Bonezzi, A. (2015). On the psychology of scarcity: When reminders of resource scarcity promote selfish (and generous) behavior. *Journal of Consumer Research, 42*(4), 615-631.

Schultz, P. W. (2002). Inclusion with nature: The psychology of human-nature relations. In *Psychology of sustainable development* (pp. 61-78): Springer.

Tan, J. J., & Kraus, M. W. (2015). Lay theories about social class buffer lower-class individuals against poor self-rated health and negative affect. *Pers Soc Psychol Bull, 41*(3), 446-461.

Liu, X. W., & Wu, J. P. (2013). Environmental values and pro-environmental behaviors of college students: Mediating role of environmental concern. *Studies of Psychology and Behavior, 11*(6), 780.
